# Unburied versus buried wires for fixation of pediatric lateral condyle distal humeral fractures: A meta-analysis

**DOI:** 10.1097/MD.0000000000007736

**Published:** 2017-08-25

**Authors:** Ya-Fei Qin, Zhi-Jun Li, Cheng-Kai Li, Shu-Cai Bai, Hui Li

**Affiliations:** Department of Orthopedics, Tianjin Medical University General Hospital, Tianjin, R.P. China.

**Keywords:** distal humeral, fractures, lateral condyle, meta-analysis

## Abstract

Open reduction and internal fixation with Kirschner (K) wires has been reported as an efficient and convenient technique for pediatric lateral condyle distal humeral fractures. However, no single study has been large enough to definitively determine whether the K-wires should be buried or unburied. Therefore, we performed a meta-analysis pooling the results from several clinical trials to compare the outcome of using buried versus unburied K-wires. Potential academic articles were identified from the Cochrane Library, Medline (1966–2017.3), PubMed (1966–2017.3), Embase (1980–2017.3), ScienceDirect (1985–2017.3), and other databases. Gray studies were identified from the references of included literature reports. RevMan 5.1 was used to analyze the pooling of data. Nonrandomized controlled trials were included in this meta-analysis. There was a significant difference in the duration of wires in situ (MD = −13.28, 95% confidence interval: −16.42 to −10.14, *P* < .00001). No significant differences were found regarding infection, superficial infection, total complications, delayed union, or reoperation. Unburied K-wire fixation for treatment of lateral condyle distal humeral fractures in children does not increase the total infection rate, superficial infection, reoperation rate, or complications. However, unburied K-wire fixation is of benefit for early extraction and impartial cost savings.

## 1 Introduction

Pediatric lateral condyle distal humeral fractures, the most common elbow fracture that involves the growth plate, account for 10% to 15% of all pediatric fractures of the elbow, with a high incidence between 4 and 10 years of age.^[1–4]^ Undisplaced fractures might be treated conservatively with casting, but there is general agreement that lateral condyle distal humeral fractures with a displacement of more than 2 mm should be treated by open reduction and internal fixation.^[5–11]^ Furthermore, Kirschner (K) wires are the most widely utilized metallic implant in displaced fractures.^[12–14]^ K-wires can be buried or left unburied outside the skin. Unburied wires can be removed in an outpatient setting, avoiding a secondary operation for wire removal. Therefore, unburied wires offer logistical and cost-saving benefits. However, it is likely that unburied wires might be more prone to pin site infection and a after deep infection.^[7]^ The duration of unburied fixation for reducing the probability of infection is about 4 weeks; however, a short duration of unburied fixation may provide inadequate time for secure union.^[15,16]^ Conversely, buried K-wires can be left in place until surgeons have explicit radiographic evidence of fracture union. Several studies comparing the outcome between buried and unburied K-wires have been published in past years.^[7,16–18]^ However, there is no clear consensus as to whether K-wires should be buried or left unburied outside the skin. Consequently, we performed a meta-analysis concerning buried or unburied internal fixation treatment of lateral condyle distal humeral fractures in children to provide evidence for making a clinical decision.

## 2 Materials and methods

### 2.1 Search strategy

**Figure 1:**
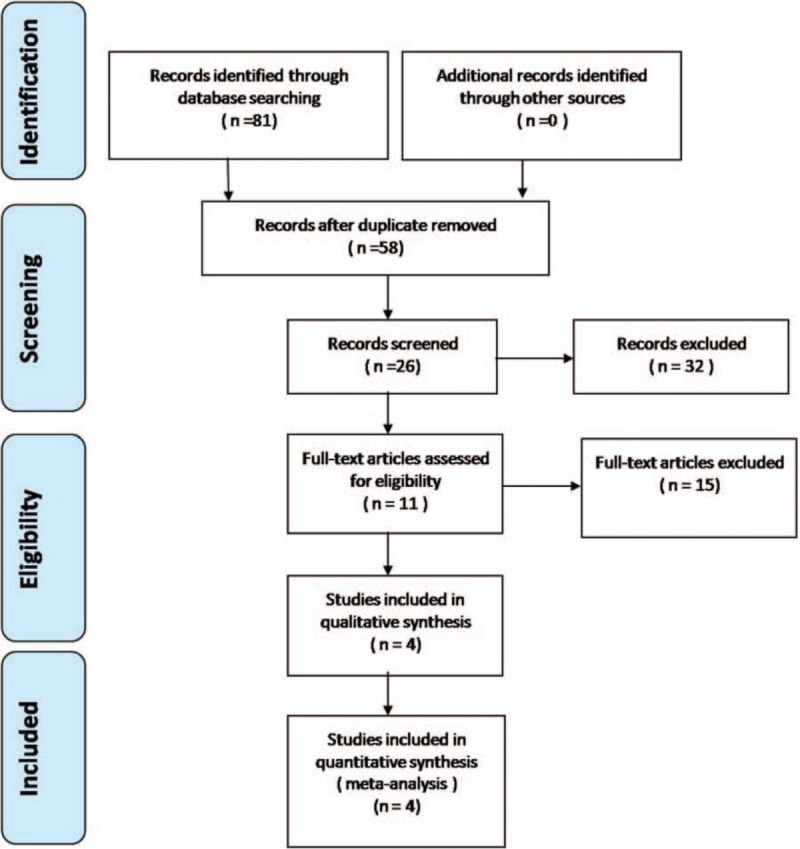
Flowchart of the study selection process.

Electronic databases including Cochrane Library, Medline (1966–2017.3), PubMed (1966–2017.3), Embase (1980–2017.3), and ScienceDirect (1985–2017.3) were searched. Gray studies were identified from the reference of included literature. No language was restricted. The search process was conducted as follows in Figure 1. The key words “lateral humeral condyle fracture,” “buried wires,” and “children or pediatric” were used in combination with the Boolean operators AND or OR. Because this was a meta-analysis, no ethics committee or institutional review board approval was required.

### 2.2 Selection criteria and quality assessment

The 2 reviewers respectively screened the retrieved literature according to the inclusion and exclusion criteria, and carried out data extraction to ensure the consistency of the results. The divergence was resolved by consultation or the third researcher. We put use of the revised general assessment tool-the Cochrane Bone, the Joint and Muscle Trauma Group, to make a quality evaluation of retrospective trials. We performed the quality assessment of randomized controlled trials (RCTs) according to the RCT bias risk evaluation tools used by the Cochrane Bone, Joint or Muscle Trauma Group. The methodological quality assessment of the included retrospective controlled were conducted by nonrandomized studies (MINORS) form for retrospective controlled trials. The methodological quality score is from 0 to 24. Disagreements were resolved by consensus or consultation with the senior reviewer.

### 2.3 Data extraction

We extracted data on the research topic, first author, publication time, average age, sex composition, inclusion and exclusion criteria, study subjects and quantity, interventions, quality control, and outcome indicators. We will consult the author without getting detailed data.

### 2.4 Data analysis and statistical methods

Statistical analyses were performed by RevMan 5.1 (The Cochrane Collaboration, Oxford, United Kingdom). Discontinuous outcomes were expressed as the risk difference (RD) with 95% confidence interval (CIs). Mean difference (MD) and 95% CIs is used for data processing of continuous outcomes, such as duration of wire usage in situ. Heterogeneity analysis was performed using the *P* values and *I*^*2*^ values in the *χ*^*2*^ test. The random effects model is applied when there is obvious heterogeneity (*I*^*2*^ > 50%) between the data. On the contrary, we use the fixed effect model when there is no heterogeneity (*I*^*2*^ < 50%) between the data.

## 3 Results

### 3.1 Search results

A total of 81 studies were identified as potential relevant literature reports. Finally, 4 literatures were included in the meta-analysis. All of them were non-RCTs and published in full text. The basic characteristics of the literatures are shown in Figure 1.

### 3.2 Risk of bias assessment

Quality assessment scores ranged from 16 to 17. For all included studies, major quality defects were inclusion of consecutive patients and less than 5% loss to follow up. More details on the quality assessment are summarized in Table 1.^[7,16–18]^

**Table 1:**
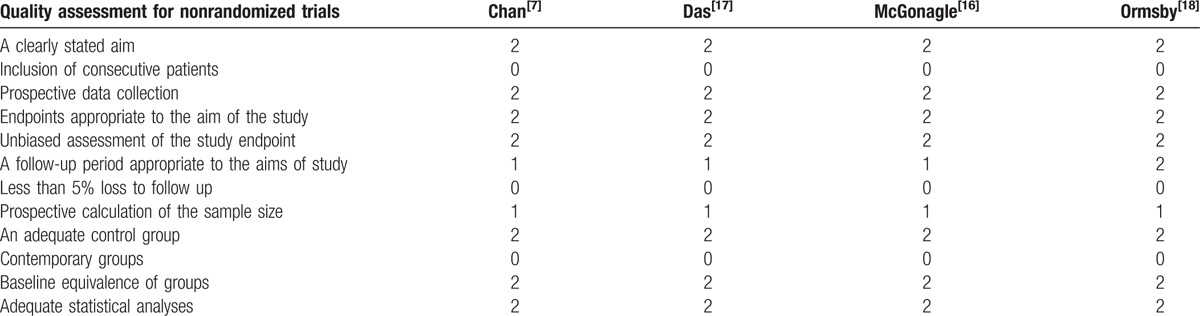
Quality assessment score of the included studies^[7,16–18]^.

### 3.3 Study characteristics

Demographic characteristics and details concerning the literature type of the included studies are summarized in Table 2.^[7,16–18]^ Statistically similar baseline characteristics were observed between both groups. All studies had small sample sizes, from 67 to 235 patients.

**Table 2:**
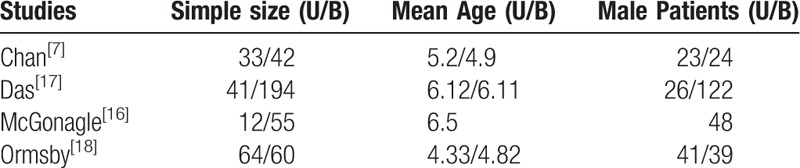
Cohort characteristics^[7,16–18]^.

### 3.4 Outcomes of meta-analysis

#### 3.4.1 Infection

**Figure 2:**
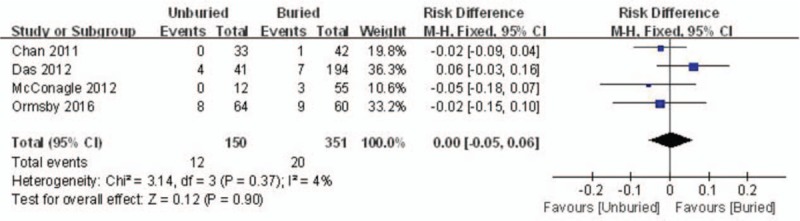
Forest plot diagram showing the incidence of infection.

The incidence of infection was provided in four reports. A fixed model was used, and no significant heterogeneity was found (*I*^*2*^ = 4%, *P* = .37). The incidence of infection in the unburied group was not higher than that in the buried group (RD = 0.00, 95% CI: −0.05 to 0.06, *P* = .90) (Fig. 2).

#### 3.4.2 Superficial infection

**Figure 3:**
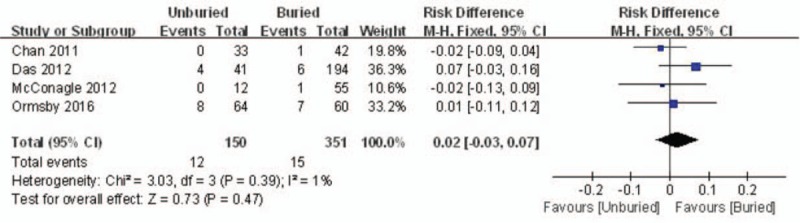
Forest plot diagram showing the incidence of superficial infection.

The incidence of superficial infection was recorded in four reports. A fixed model was used, and no significant heterogeneity was found (*I*^*2*^ = 1%, *P* = .39). The incidence of superficial infection in the unburied group was not higher than that in the buried group (RD = 0.02, 95% CI: −0.03 to 0.07, *P* = .47) (Fig. 3).

#### 3.4.3 Duration of wire usage in situ

**Figure 4:**
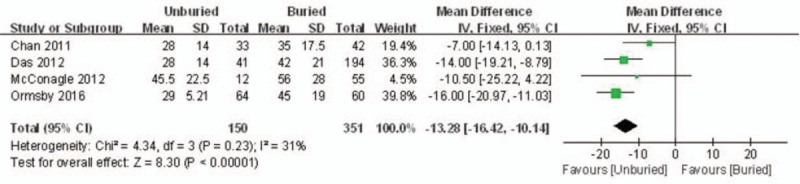
Forest plot diagram showing the duration of wires in situ.

The duration of the usage of wires in situ was reported in 4 published works. Applying a fixed model, significant heterogeneity was found (*I*^*2*^ = 31%, *P* = .23). The duration of wire usage in situ in the unburied group was significantly lower than that in the buried group (MD = −13.28, 95% CI: −16.42 to −10.14, *P* < .00001) (Fig. 4).

#### 3.4.4 Reoperation

**Figure 5:**
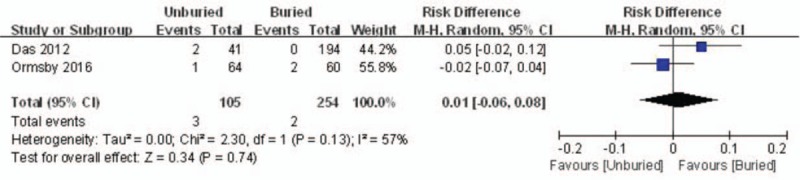
Forest plot diagram showing the incidence of reoperation.

Two studies reported the incidence rate of reoperation. There was significant heterogeneity (*I*^*2*^ = 75%, *P* = .05), therefore, a random-model was performed. Pooling results demonstrated that the incidence of re-operation in the unburied group was not significantly higher than that in the buried group (MD = 0.01, 95% CI: −0.06 to 0.08, *P* = .74) (Fig. 5).

#### 3.4.5 Delayed union

**Figure 6:**
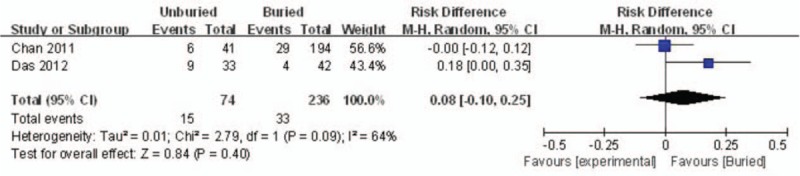
Forest plot diagram showing the incidence of delayed union.

Two articles reported the incidence rate of delayed union. A random model was used because of significant heterogeneity (*I*^*2*^ = 64%, *P* = .09). No significant difference between the 2 groups was detected (RD = 0.08, 95% CI: −0.10 to 0.25, *P* = .40) (Fig. 6).

#### 3.4.6 Total complications

**Figure 7:**
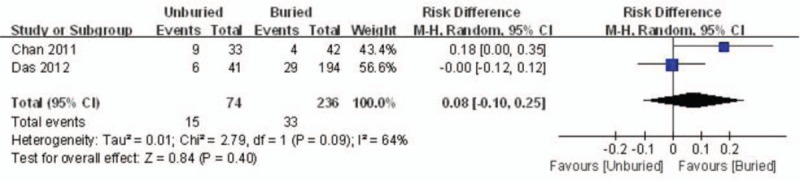
Forest plot diagram showing the total complications.

Total complications were reported in 2 studies. Significant heterogeneity was found; thus, a random model was used (*I*^*2*^ = 64%, *P* = .09). There was no significant difference between the 2 groups regarding the incidence rate of total complications (RD = 0.08, 95% CI: −0.10 to 0.25, *P* = .40) (Fig. 7).

## 4 Discussion

Lateral condyle distal humeral fractures are intra-articular fractures (Milch type II) according to the Salter-Harris classification. The fractures have a high incidence rate of complications including nonunion, malunion, ulnar nerve paresis, and angular deformity.^[19–22]^ The micromovement of the fracture site caused by the muscular extension of the wrist can lead to insufficient fracture healing and internal fixation. Therefore, in order to reduce the incidence of complications, people have a precise anatomical and stable fixation for the pediatric humeral lateral condylar fractures.^[23]^ Therefore, lateral condyle distal humeral fractures in children require accurate anatomical reduction and stable fixation to minimize complications. Clinical studies have reported on various technical aspects of fixation for lateral condyle distal humeral fractures.^[14,24–27]^ Closed reduction and K-wire fixation are recommend for lateral condyle distal humeral fractures in children, according to the research of Song et al.^[28]^ However, Gaston^[29]^ holds the point of view that closed reduction could increase the risk of complications, though some researchers demonstrate that screw fixation could promote the union of fracture without significant complications.^[14,24,30,31]^ The use of K-wires is still the most frequently used technique in clinical work. However, there is currently no consensus as to whether the K-wires could be buried or left unburied outside the skin.

One of the obvious advantages of buried K-wires is to avoid external contact and reduce postoperative infection. However, buried K-wires have the potential to erode and penetrate the skin as the swelling subsides. Our study indicates that buried K-wires have a higher risk of complications of skin erosion and deep infection, which is in consistence with other clinical research. The incidence rate of buried K-wires protruding through the skin was 16% in a retrospective study of 235 cases of children with humeral condylar fractures.^[17]^ On the other hand, the main benefit of buried wires is that the wires can be left in situ until the surgeons have clinical and radiographic evidence of fracture union. However, there is no significant difference between the use of buried and unburied K-wires in the reoperation rate or the delayed union rate, which is consistent with McGonagle's clinical research.^[16]^

In our study, Das De and Ormsby^[17,18]^ report that unburied wires would evidently impart cost-savings compared with buried wires. Higher postoperative infection occurred among the 56 patients with distal radius fractures in the unburied K-wire group according to Hargreaves’^[32]^ prospective randomized study, and he recommends a buried fixation duration of more than 8 weeks in spite of higher cost. Our research demonstrates that there is no significant difference in the postoperative infection rate or superficial infection between the buried and unburied groups, but the unburied group needs less time for removing K-wires. Furthermore, in comparing 100 lateral condyle distal humeral fractures in children, Thomas^[33]^ believes that a basic healing can be achieved 3 weeks after the operation.

Chan^[7]^ describe 2 fixation techniques in a retrospective controlled study. They explain the reasons for 2 different approaches: buried wires bend close to the bone and the bending of the wires prevents the fragmentation from sliding along the wires and shifting it. The unburied wires are not bent close to the bone. Therefore, it is generally considered that the wider divergence angle and an additional wire are needed to provide sufficient stability. An effective treatment with short-term oral antibiotics was performed in one case of needle tract infection (3%), and silver nitrate treated two patients with hypergranulation (7%) of needle tract. In addition, no disunion, deformity, or reoperation occurred in either group. In addition, there was no occurrence of nonunion, malunion, or reoperation in either of the groups. Thus, we believe the outcomes of both surgical techniques were adequate for stable fixation, with no serious complications.

Infection is one of the most common complications in the treatment of pediatric lateral condylar fractures with K-wire. Four included studies found that the incidence of infection in the unburied group was not higher than that in the buried group, which is consistent with our meta-analysis. Three included articles reported that the K-wires were removed at 4 weeks, whereas McGonagall removed all K-wires after an average of 6.5 weeks (range: 5.0–8.7 weeks). There is some controversy regarding the timing of the removal of wires in the literature. However, Chan^[7]^ recommends a protocol involving removal at 4 weeks, with 2 weeks of subsequent immobilization in a backslab, to be safe and effective, regardless of radiological evidence of a callus.

For published studies with small samples, we searched and included all randomized controlled trials (RCTs) and nonrandomized controlled trials (non-RCTs). However, some clinical studies did not provide statistical data. Four non-RCTs^[7,16–18]^ ultimately met the inclusion criteria for the meta-analysis without high quality RCTs. All these shortcomings weaken the level of evidence for the current study.

## 5 Conclusion

The use of unburied K-wire fixation for treatment of lateral condyle distal humeral fracture in children does not increase the total infection rate, superficial infection rate, reoperation rate, or complications, but use of unburied K-wire fixation has the benefit of early extraction and impartial cost savings. Large sample sizes, long-term follow up, and well-designed studies are needed in the future.
